# Cabozantinib in Japanese patients with advanced hepatocellular carcinoma: a phase 2 multicenter study

**DOI:** 10.1007/s00535-020-01753-0

**Published:** 2021-01-03

**Authors:** Masatoshi Kudo, Kaoru Tsuchiya, Naoya Kato, Atsushi Hagihara, Kazushi Numata, Hiroshi Aikata, Yoshitaka Inaba, Shunsuke Kondo, Kenta Motomura, Junji Furuse, Masafumi Ikeda, Manabu Morimoto, Meguru Achira, Shingo Kuroda, Akiko Kimura

**Affiliations:** 1 Department of Gastroenterology and Hepatology, Faculty of Medicine, Kindai University, 377-2 Ohno-Higashi, Osaka-Sayama, 589-8511 Japan; 2 Department of Gastroenterology and Hepatology, Musashino Red Cross Hospital, Tokyo, Japan; 3 Department of Gastroenterology, Graduate School of Medicine, Chiba University, Chiba, Japan; 4 Department of Hepatology, Graduate School of Medicine, Osaka City University, Osaka, Japan; 5 Gastroenterological Center, Yokohama City University Medical Center, Yokohama, Japan; 6 Department of Gastroenterology and Metabolism, Graduate School of Biomedical and Health Sciences, Hiroshima University, Hiroshima, Japan; 7 Department of Diagnostic and Interventional Radiology, Aichi Cancer Center Hospital, Nagoya, Japan; 8 Department of Hepatobiliary and Pancreatic Oncology, National Cancer Center Hospital, Tokyo, Japan; 9 Department of Hepatology, IIZUKA HOSPITAL, Iizuka, Japan; 10 Department of Medical Oncology, Faculty of Medicine, Kyorin University, Tokyo, Japan; 11 Department of Hepatobiliary and Pancreatic Oncology, National Cancer Center Hospital East, Kashiwa, Chiba Japan; 12 Department of Hepatobiliary Pancreatic Oncology, Kanagawa Cancer Center, Kanagawa, Japan; 13 Clinical Pharmacology, PRA Development Center KK, Osaka, Japan; 14 Biostatistics, Takeda Pharmaceutical Company Limited, Osaka, Japan; 15 Oncology Clinical Research Department, Oncology Therapeutic Area Unit for Japan and Asia, Takeda Pharmaceutical Company Limited, Osaka, Japan

**Keywords:** Hepatocellular carcinoma, Cabozantinib, Sorafenib, Lenvatinib, Japan

## Abstract

**Background:**

To evaluate the efficacy and safety of cabozantinib in Japanese patients with advanced hepatocellular carcinoma (HCC) who had progressed following one or two lines of systemic therapy including sorafenib. An exploratory evaluation in sorafenib-naïve patients was performed.

**Methods:**

In this open-label, single-arm, phase 2 trial, patients received oral cabozantinib 60 mg once daily. The primary endpoint was progression-free survival (PFS) rate at Week 24. Secondary endpoints included PFS, overall survival (OS), objective response rate (ORR, best response of complete/partial response), disease control rate (DCR, objective response or stable disease) and safety.

**Results:**

Thirty-four patients received cabozantinib across 17 centers (prior sorafenib cohort, *n* = 20; sorafenib-naïve cohort, *n* = 14). PFS rate at 24 weeks was 59.8% [90% confidence interval (CI) 36.1–77.2%] in the prior sorafenib cohort, 16.7% (90% CI 4.0–36.8%) in the sorafenib-naïve cohort and 40.1% (90% CI 24.8–55.0%) overall. Median PFS was 7.4 months for the prior sorafenib cohort, 3.6 months for the sorafenib-naïve cohort, and 5.6 months overall. OS rate at 6 months was 100.0%, 78.6% and 91.1%, respectively; DCR was 85.0%, 64.3% and 76.5%, respectively. The ORR was 0.0% for both cohorts. All patients required dose modifications due to adverse events, the most common of these were palmar–plantar erythrodysesthesia syndrome and diarrhea. Three patients (8.8%) discontinued due to adverse events other than disease progression.

**Conclusions:**

Cabozantinib 60 mg/day has a favorable benefit/risk profile for Japanese patients with advanced HCC who have previously received one or two lines of systemic anticancer therapy including sorafenib. (Clinical trial registration: NCT03586973)

**Supplementary Information:**

The online version contains supplementary material available at 10.1007/s00535-020-01753-0.

## Introduction

Liver cancer is the fifth-highest cause of cancer-related mortality in Japan, accounting for a total of 40,099 deaths in 2014 [1]. Approximately 90% of all liver cancers are hepatocellular carcinomas (HCC), and HCC is a notoriously chemo-resistant tumor type [2, 3]. Patients with localized HCC can undergo curative resection or other regional therapy (local ablation, chemoembolization or other transcatheter therapies), but those who present with advanced, unresectable disease have a poor prognosis [4].

Since 2007, standard of care first-line therapy for advanced, unresectable HCC has been systemic treatment with sorafenib monotherapy [4]. Both the SHARP and Asia Pacific phase 3 trials showed a significant survival benefit with sorafenib versus placebo in this population, with an approximate 32% reduction in the relative risk of death [5, 6]. Survival benefits associated with sorafenib were achieved without notable tumor shrinkage as indicated by objective tumor response rates (ORR) of 2–3% [5, 6]. More recently, the REFLECT phase 3 study demonstrated non-inferiority for lenvatinib compared with sorafenib in terms of overall survival (OS) as first-line therapy for advanced HCC [7]. Nevertheless, the majority of patients with unresectable disease will progress on first-line therapy and require second-line treatment.

Cabozantinib is an oral inhibitor of vascular endothelial growth factor (VEGF) receptors 1, 2, and 3, MET and AXL [8, 9]. By inhibiting MET and AXL, cabozantinib targets oncogenic and angiogenic pathways that may provide additional efficacy to help overcome resistance to agents targeting the VEGF receptor pathway [10]. In particular, MET expression has been shown to be increased in patients with HCC previously exposed to sorafenib and may represent a mechanism of sorafenib resistance [11, 12]. The CELESTIAL phase 3 trial demonstrated a significant survival benefit with cabozantinib versus placebo in patients previously treated with sorafenib as indicated by a 24% reduction in relative risk of death [13]. Cabozantinib has since been approved in the United States and Europe for patients with HCC previously treated with sorafenib.

The CELESTIAL study population included patients from the Asia–Pacific region, Europe and North America, but did not include patients from Japanese clinical centers [13]. The aim of this phase 2 study was to evaluate the efficacy and safety of cabozantinib in Japanese patients with advanced HCC who had received prior sorafenib, including patients who were intolerant to sorafenib. The study was also designed to take into account recent changes to advanced HCC clinical care in Japan following approval of a number of other systemic therapies, including lenvatinib, and second-line treatment options regorafenib and ramucirumab [14, 15]. Therefore, a second cohort of patients was included who had received prior systemic therapy for advanced HCC that did not include sorafenib to be assessed in an exploratory manner.

## Methods

### Study design and treatment

This was an open-label, single-arm, multicenter, phase 2 study designed to evaluate the efficacy and safety of cabozantinib in Japanese patients with advanced HCC who had received one or two lines of prior systemic anticancer therapy (Clinical trial registration number: NCT03586973). Enrolled patients were entered into one of two study cohorts depending on whether their treatment history included sorafenib (prior sorafenib cohort or sorafenib-naïve cohort). The primary objective was to assess the efficacy of cabozantinib via the rate of progression-free survival (PFS) at 24 weeks for patients who had received prior sorafenib (prior sorafenib cohort).

Cabozantinib 60 mg was administered orally, in tablet form, once daily (QD) in the fasted state (≥ 2 h after a meal, with no food ingested for ≥ 1 h after dosing), preferably at bedtime. Study drug dose interruptions and dose reductions (to 40 mg QD or 20 mg QD) were permitted to manage adverse events (AEs). Patients continued to receive study treatment if they experienced clinical benefit in the opinion of the investigator.

Written informed consent was obtained from each patient prior to screening. The study protocol, amendments, informed consent and other related documents were reviewed and approved by each center’s Institutional Review Board, and the study was conducted according to the International Council for Harmonization Good Clinical Practice guidelines and the ethical principles of the Declaration of Helsinki.

### Patients

Eligible patients were Japanese, aged ≥ 20 years, with a histological or cytological diagnosis of HCC, with measurable disease by Response Evaluation Criteria in Solid Tumors version 1.1 (RECIST v1.1) that was not amenable to curative treatment. Patients had received one or two prior anticancer therapies for advanced HCC with subsequent radiographic progression. Patients were also required to have a Child–Pugh liver function class A, an Eastern Cooperative Oncology Group (ECOG) performance status of 0 or 1 and a life expectancy ≥ 3 months. Exclusion criteria included any type of anticancer agent < 14 days prior to study drug initiation, any radiotherapy < 28 days prior (< 14 days for radiotherapy of bone metastases) or radionuclide treatment < 42 days prior. Patients with fibrolamellar carcinoma or mixed hepatocellular cholangiocarcinoma were also excluded.

### Endpoints and assessments

The primary endpoint was PFS rate at 24 weeks, defined as the proportion of patients who were alive and without progressive disease (PD) at Week 25 Day 1 of the study treatment period. Secondary endpoints included PFS, OS, ORR [defined as the proportion of patients with a best overall tumor response of complete response (CR) or partial response (PR)], disease control rate [DCR, defined as the proportion of patients with a CR, PR or stable disease (SD)], pharmacokinetics and safety. Radiographic response and disease progression were determined by a central, blinded Independent Radiology Committee using RECIST v1.1 as in the CELESTIAL study [13]. Blood samples were obtained from all patients to measure cabozantinib plasma concentration at pre-specified time points, including predose at Week 3 Day  1, Week 5 Day 1 and Week 9 Day 1. Safety was evaluated continuously throughout the study and up to 30 days after the last dose of study drug. Predefined AEs of special interest (AESIs) related to antiangiogenic therapy were recorded. AEs were coded using the Medical Dictionary for Regulatory Activities (MedDRA) version 22.0 and AE severity was determined using National Cancer Institute Common Terminology Criteria for AEs version 4.03.

### Statistical analysis

In the CELESTIAL study of subjects with advanced HCC who had received prior sorafenib, the 24 week PFS rate in the cabozantinib group was 38.4% [95% confidence interval (CI) 33.5–43.3%] vs. 11.1% (95% CI 7.2–15.8%) in the placebo group (data not published) [13]. Based on these data, a sample size of 17 patients in the prior sorafenib cohort of this study was estimated to provide ≥ 80% probability that the lower limit of the 2-sided 90% CI for the 24 week PFS rate would be ≥ 11.1%, when assuming the true 24-week PFS rate to be ≥ 38.4%. A total study population of approximately 32 patients was planned, including a sorafenib-naïve cohort, to provide additional insight into the safety and efficacy of cabozantinib in a Japanese HCC population.

Efficacy and safety were assessed in all patients who received at least one dose of study drug. The primary endpoint was evaluated by Kaplan–Meier estimates and corresponding 90% CIs, calculated using the Greenwood’s formula and complementary log–log transformation.

## Results

**Fig. 1:**
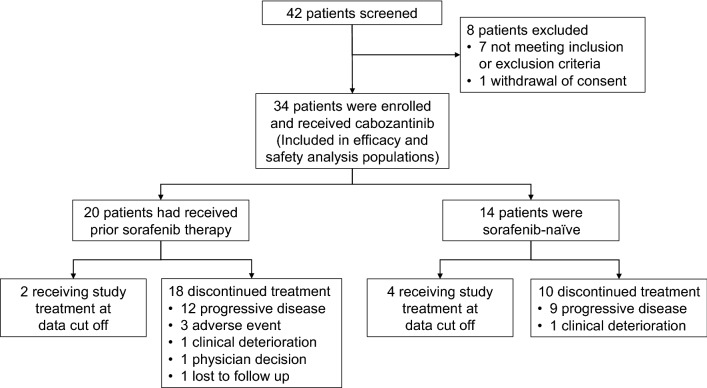
Patient disposition (CONSORT flowchart)

The study was conducted between August 2018 and the data cutoff in July 2019 at 17 Japanese clinical centers. A total of 42 patients with advanced HCC were assessed for eligibility, of whom eight were excluded during screening (Fig. 1). The remaining 34 patients were enrolled and received study treatment. Of the 34 enrolled, 20 patients had received prior sorafenib therapy for advanced HCC (prior sorafenib cohort) and 14 patients had not previously received sorafenib (sorafenib-naïve cohort). All 34 patients were included in the efficacy and safety analysis populations. At the data cutoff, study treatment had been discontinued by 90.0% (18/20) of patients in the prior sorafenib cohort and 71.4% (10/14) in the sorafenib-naïve cohort. Progressive disease was the most common reason for discontinuation.

Baseline demographic and clinical characteristics are summarized in Table 1. Approximately 40% of patients in both cohorts had extrahepatic spread, no patient had macrovascular invasion, and the majority of patients had baseline alpha-fetoprotein (AFP) levels < 400 ng/mL (70.6%, 24/34). Most patients (76.5%, 26/34) had received one prior systemic anticancer regimen for advanced HCC, with the remaining patients (23.5%, 8/34) having received two prior regimens. Median (range) duration of sorafenib therapy in the prior sorafenib cohort was 7.9 (0.4–24.0) months and five patients (25.0%, 5/20) had discontinued sorafenib therapy due to intolerance (Supplementary Table 1). Two (10%) patients in the prior sorafenib cohort received prior lenvatinib with a median treatment duration of 2.2 (0.1–4.4) months and had discontinued lenvatinib due to intolerance (*n* = 1) or disease progression (*n* = 1). Ten (71.4%) patients in the sorafenib-naive cohort received prior lenvatinib with a median treatment duration of 3.5 (1.3–6.5) months, and discontinued lenvatinib due to disease progression in all 10 cases.

**Table 1:** Demographics and baseline characteristics (full analysis set)

	Prior sorafenib (*n* = 20)	Sorafenib-naïve (*n* = 14)	Total (*n* = 34)
Median age, years (range)	73.0 (59, 82)	73.0 (55, 81)	73.0 (55, 82)
< 65 years (%)	2 (10.0)	3 (21.4)	5 (14.7)
65 to < 75 years (%)	11 (55.0)	4 (28.6)	15 (44.1)
≥ 75 years (%)	7 (35.0)	7 (50.0)	14 (41.2)
Sex, *n* (%)			
Male	17 (85.0)	14 (100.0)	31 (91.2)
Female	3 (15.0)	0 (0.0)	3 (8.8)
ECOG PS score, *n* (%)			
0	20 (100.0)	11 (78.6)	31 (91.2)
1	0 (0.0)	3 (21.4)	3 (8.8)
Etiology of HCC, *n* (%)*			
HBV	5 (25.0)	2 (14.3)	7 (20.6)
HCV	7 (35.0)	4 (28.6)	11 (32.4)
Dual HBV and HCV	0 (0.0)	1 (7.1)	1 (2.9)
Alcoholism	5 (25.0)	5 (35.7)	10 (29.4)
Nonalcoholic steatohepatitis	1 (5.0)	2 (14.3)	3 (8.8)
Other	3 (15.0)	2 (14.3)	5 (14.7)
ALBI grade, *n* (%)			
1	11 (55.0)	7 (50.0)	18 (52.9)
2	9 (45.0)	7 (50.0)	16 (47.1)
3	0 (0.0)	0 (0.0)	0 (0.0)
Child–Pugh score, *n* (%)			
A	20 (100.0)	14 (100.0)	34 (100.0)
Current extent of disease, *n* (%)			
Macrovascular invasion	0 (0.0)	0 (0.0)	0 (0.0)
Extrahepatic spread	7 (35.0)	6 (42.9)	13 (38.2)
No. of involved organs^†^, *n* (%)			
0	1 (5.0)	0 (0.0)	1 (2.9)
1	13 (65.0)	8 (57.1)	21 (61.8)
2	3 (15.0)	6 (42.9)	9 (26.5)
≥ 3	3 (15.0)	0 (0.0)	3 (8.8)
AFP (ng/mL)			
Median (range)	51.5 (2.0–21,300)	148.5 (2.1–8990)	63.8 (2.0–21,300)
< 400 ng/mL, *n* (%)	16 (80.0)	8 (57.1)	24 (70.6)
≥ 400 ng/mL, *n* (%)	4 (20.0)	6 (42.9)	10 (29.4)
No. of prior systemic non-radiation anticancer agents for HCC, *n* (%)			
1	12 (60.0)	10 (71.4)	22 (64.7)
2	8 (40.0)	4 (28.6)	12 (35.3)
Prior systemic anticancer agent for HCC, *n* (%)			
Sorafenib	20 (100.0)	0	20 (58.8)
Lenvatinib	2 (10.0)	10 (71.4)	12 (35.3)
Agent targeting PD-1, PD-L1	1 (5.0)	4 (28.6)	5 (14.7)

*AFP* alpha-fetoprotein, *ALBI* albumin-bilirubin, *ECOG * Eastern Cooperative Oncology Group performance status, *HBV* hepatitis B virus, *HCC* hepatocellular carcinoma, *HCV* hepatitis C virus, *PD-1* programmed cell death protein 1, *PD-L1/L2* programmed cell death-ligand 1/2

*Two patients had multiple HCC etiologies: one patient in the prior sorafenib cohort (HBV and alcoholism) and one in the sorafenib-naïve cohort (HBV and HCV)

^†^As determined by an independent radiology committee

### Efficacy

**Fig. 2:**
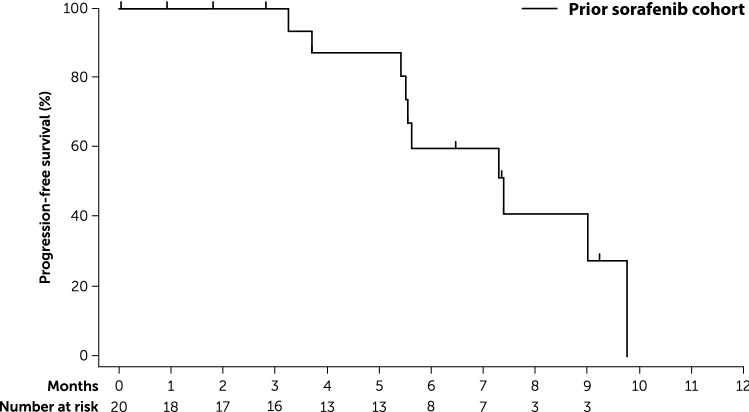
Kaplan–Meier analysis of progression-free survival in patients with prior sorafenib exposure (full analysis set)

**Fig. 3:**
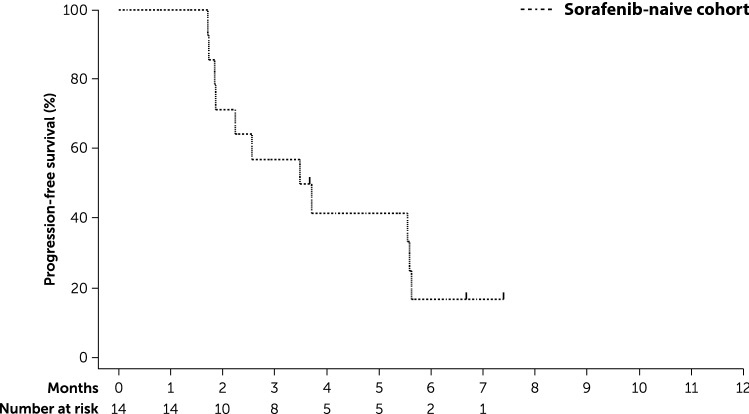
Kaplan–Meier analysis of progression-free survival in sorafenib-naïve patients (full analysis set)

The primary endpoint of PFS rate at 24 weeks was 59.8% (90% CI 36.1–77.2) for the prior sorafenib cohort, 16.7% (90% CI 4.0–36.8) for the sorafenib-naïve cohort, and 40.1% (90% CI 24.8–55.0) for the total study population. In the prior sorafenib cohort, the lower limit of the 90% CI exceeded the prespecified threshold of 11.1%. Median PFS was 7.4 months (95% CI 5.5–9.8) in the prior sorafenib cohort (Fig. 2), 3.6 months (95% CI 1.8–5.6) in the sorafenib-naïve cohort (Fig. 3), and 5.6 months (95% CI 3.7–7.4) for the total study population.

OS data were not fully mature at data cutoff, with a follow-up time from the enrollment of the last patient to data cutoff of approximately 8 months for the prior sorafenib cohort and 6 months for the sorafenib-naïve cohort (Supplementary Fig. 1). The estimated OS rate at 6 months was 100.0% (95% CI 100.0–100.0) for the prior sorafenib cohort, 78.6% (95% CI 47.3–92.5) for the sorafenib-naïve cohort and 91.1% (95% CI 74.8–97.0) for the total study population. Median OS in the prior sorafenib cohort and the overall population was 10.9 months (95% CI 9.8–10.9) but was not reached in the sorafenib-naïve cohort.

No patients achieved a CR or PR, and the ORR for both cohorts was, therefore, 0.0% (Table 2). DCR was achieved by 85.0% (17/20) in the prior sorafenib cohort, 64.3% (9/14) in the sorafenib-naïve cohort, and 76.5% (26/34) patients overall. A ≥ 50% reduction from baseline in AFP levels was achieved by 15.0% (3/20) of patients in the prior sorafenib cohort and none in the sorafenib-naïve cohort.

**Table 2:** Tumor response rates (full analysis set)

	Prior sorafenib (*n* = 20)	Sorafenib-naïve (*n* = 14)	Total (*n* = 34)
Best overall response, *n* (%)*			
CR	0	0	0
PR	0	0	0
SD	17 (85.0)	9 (64.3)	26 (76.5)
PD	1 (5.0)	4 (28.6)	5 (14.7)
ORR, *n* (%)			
CR + PR	0	0	0
95% CIs	0–16.8	0–23.2	0–10.3
DCR, *n* (%)			
CR + PR + SD	17 (85.0)	9 (64.3)	26 (76.5)
95% CIs	62.1–96.8	35.1–87.2	58.5–89.3

*CIs* confidence intervals, *CR* complete response, *DCR* disease control rate, *ORR* overall response rate, *PD* progressive disease, *PR* partial response, *SD* stable disease

*Tumor response data were missing for two patients in the prior sorafenib cohort and one patient in the sorafenib-naïve cohort

### Pharmacokinetics

Cabozantinib plasma concentrations generally decreased over the course of the study in line with dose modifications. In the prior sorafenib cohort, median (range) cabozantinib plasma concentrations were 2155 (1120–3440) ng/mL (*n* = 20) at Week 3, 1505 (459–3110) ng/mL (*n* = 18) at Week 5, and 961 (8.53–2530) ng/mL (*n* = 17), at Week 9. Similarly, in the sorafenib-naïve cohort, values were 2110 (24.4–3570) ng/mL (*n* = 14) at Week 3, 1705 (134–3450) ng/mL (*n* = 12) at Week 5, and 495 (11.6–1830) ng/mL (*n* = 12) at Week 9.

### Safety

The median (range) duration of study drug exposure was 7.4 (0.9–9.3) months for the prior sorafenib cohort with a median dose intensity of 38.7%; for the sorafenib-naïve cohort, the values were 4.8 (0.8–8.9) months and 33.7%, respectively (Table 3). Dose reductions due to an AE occurred in 95.0% (19/20) of patients in the prior sorafenib cohort, 85.7% (12/14) in the sorafenib-naïve cohort and 91.2% (31/34) of patients overall. The most frequent AEs leading to dose reduction across the total population were palmar–plantar erythrodysesthesia (PPE) syndrome (35.3%, 12/34) and diarrhea (14.7%, 5/34). AEs leading to study drug discontinuation, excluding AEs of disease progression, occurred in 8.8% of patients (3/34; all in the prior sorafenib cohort) and comprised two cases of PPE syndrome and one case each of gastrointestinal ulcer and malaise.

**Table 3:** Study drug dosing intensity and modification (safety analysis set)

	Prior sorafenib (*n* = 20)	Sorafenib-naïve (*n* = 14)	Total (*n* = 34)
Median duration of study drug exposure, months (range)	7.4 (0.9–9.3)	4.8 (0.8–8.9)	5.6 (0.8–9.3)
Median dose intensity, % (range)	38.7 (13.1–64.3)	33.7 (20.8–74.8)	37.3 (13.1–74.8)
Patients with a dose reduction due to an AE, *n* (%)	19 (95.0)	12 (85.7)	31 (91.2)
Median time to 1st dose reduction due to an AE, days (range)*	29.0 (3–58)	29.0 (10–58)	29.0 (3–58)
Median time to 2nd dose reduction due to an AE, days (range)^†^	85.0 (8–197)	59.0 (15–141)	75.5 (8–197)
Patients with a dose interruption due to an AE, *n* (%)	18 (90.0)	13 (92.9)	31 (91.2)
Median time to 1st dose interruption due to AE, days (range)^‡^	27.5 (2–197)	21.0 (6–36)	22.0 (2–197)
Median time to 2nd dose interruption due to AE, days (range)^§^	68.0 (20–246)	92.0 (48–197)	69.5 (20–246)

*n = 19, 12 and 31 in in the prior sorafenib, sorafenib-naïve and total population cohorts, respectively

^†^*n* = 17, 9 and 26 in the prior sorafenib, sorafenib-naïve and total population cohorts, respectively

^‡^*n* = 18, 13 and 31 in the prior sorafenib, sorafenib-naïve and total population cohorts, respectively

^§^*n* = 13, 11 and 24 in the prior sorafenib, sorafenib-naïve and total population cohorts, respectively

All patients experienced at least one AE of any grade, and AEs of grade 3 or 4 were reported for 79.4% (27/34) of patients (Table 4). The most frequent AEs of any grade were PPE syndrome (76.5%, 26/34), diarrhea (61.8%, 21/34) and hypertension (47.1%, 16/34). The most frequent grade 3 or 4 AEs were PPE syndrome (26.5%, 9/34), hypertension (23.5%, 8/34) and neutrophil count decreased (11.8%, 4/34). The incidence of grade 4 AEs was 11.8% (4/34), comprising two patients with lipase increased and one patient each with bacterial meningitis, and hypocalcemia and hypomagnesemia. No grade 5 AEs were reported. The incidence of serious AEs was 26.5% (9/34). Cholangitis was reported in two patients (one in each cohort), with all other events reported in one patient. Serious AEs for which a causal relationship to study drug could not be ruled out occurred in 14.7% of patients (5/34), with one case each of gastrointestinal ulcer, ileus, bacterial peritonitis, and hypocalcemia in the prior sorafenib cohort, and one case of hepatic encephalopathy in the sorafenib-naïve cohort. Six deaths were reported, all of which occurred > 30 days after the last study drug dose and were attributed to disease progression.

**Table 4:** Summary of treatment-emergent adverse events occurring in ≥ 20% of the total population (safety analysis set)

*n* (%)	Prior sorafenib (*n* = 20)		Sorafenib-naïve (*n* = 14)		Total (*n* = 34)	
	Any grade	Grade ≥ 3	Any grade	Grade ≥ 3	Any grade	Grade ≥ 3
Any TEAE	20 (100.0)	16 (80.0)	14 (100.0)	11 (78.6)	34 (100.0)	27 (79.4)
Palmar-plantar erythrodysethesia syndrome	16 (80.0)	4 (20.0)	10 (71.4)	5 (35.7)	26 (76.5)	9 (26.5)
Diarrhea	15 (75.0)	1 (5.0)	6 (42.9)	0	21 (61.8)	1 (2.9)
Hypertension	9 (45.0)	4 (20.0)	7 (50.0)	4 (28.6)	16 (47.1)	8 (23.5)
Decreased appetite	8 (40.0)	0	7 (50.0)	0	15 (44.1)	0
Platelet count decreased	6 (30.0)	0	7 (50.0)	3 (21.4)	13 (38.2)	3 (8.8)
AST increased	6 (30.0)	0	6 (42.9)	0	12 (35.3)	0
ALT increased	5 (25.0)	0	5 (35.7)	0	10 (29.4)	0
Hypothyroidism	7 (35.0)	0	3 (21.4)	0	10 (29.4)	0
Dysphonia	8 (40.0)	0	0	0	8 (23.5)	0
Fatigue	5 (25.0)	0	3 (21.4)	1 (7.1)	8 (23.5)	1 (2.9)
Malaise	6 (30.0)	0	2 (14.3)	0	8 (23.5)	0
Dysgeusia	4 (20.0)	0	3 (21.4)	0	7 (20.6)	0
Neutrophil count decreased	5 (25.0)	4 (20.0)	2 (14.3)	0	7 (20.6)	4 (11.8)
Proteinuria	3 (15.0)	2 (10.0)	4 (28.6)	1 (7.1)	7 (20.6)	3 (8.8)
Pyrexia	3 (15.0)	0	4 (28.6)	0	7 (20.6)	0
Rash	3 (15.0)	0	4 (28.6)	0	7 (20.6)	0

*ALT* alanine aminotransferase, *AST* aspartate aminotransferase, *NA* not applicable, *TEAE* treatment-emergent adverse event

The most common (> 10%) AESIs of any grade in the total population were PPE syndrome (76.5%, 26/34), diarrhea (61.8%, 21/34), hypertension (47.1%, 16/34), hypothyroidism (29.4%, 10/34), proteinuria (20.6%, 7/34) and hepatotoxicity (11.8%, 4/34) (Supplementary Table 2). Two patients in the prior sorafenib cohort discontinued study treatment due to an AESIs (PPE syndrome in both cases). Reported serious AESIs were bacterial peritonitis (gastrointestinal perforation), liver abscess (intra-abdominal and pelvic abscess), and hepatic encephalopathy (hepatotoxicity; 1 patient). No patient had an AESI of fistula, hemorrhage (≥ Grade 3), wound complication, osteonecrosis, reversible posterior leukoencephalopathy syndrome, or QT prolongation.

## Discussion

The results of this study indicate that cabozantinib therapy for advanced HCC has comparable effectiveness in Japanese patients previously treated with sorafenib as was previously observed in the non-Japanese population of the CELESTIAL study, and with no new safety concerns [13]. Furthermore, our exploratory analysis showed promising efficacy for cabozantinib in patients with advanced HCC who had received prior therapy other than sorafenib.

The CELESTIAL study established the efficacy of cabozantinib in patients with HCC previously treated with sorafenib, showing a significant increase versus placebo in OS [10.2 months (95% CI 9.1–12.0) vs. 8.0 months (95% CI 6.8–9.4)] and PFS [5.2 months (95% CI 4.0–5.5) vs. 1.9 months (95% CI 1.9–1.9)] [13]. Subgroup analysis of PFS uniformly favored cabozantinib over placebo across all subgroups with ≥ 20 patients [13]. PFS rate at 24 weeks was selected as the primary endpoint in this study (rather than ORR typical of other phase 2 studies) because the CELESTIAL study showed that the survival benefit of cabozantinib was not associated with marked tumor shrinkage by RECIST v.1.1. Both the PFS rate at 24 weeks and the estimated OS rate at 6 months in the prior sorafenib cohort of this study were higher than those in the CELESTIAL study (59.8% vs. 38.4% and 100% vs. 72%) [13]. Patients in the cabozantinib arm of the CELESTIAL study might have had more aggressive HCC and a poorer prognosis at baseline than those in the prior sorafenib cohort of this study, as reflected by a higher rate of macrovascular invasion (27% vs 0%), an ECOG performance status > 0 (48% vs 0%) and AFP level ≥ 400 ng/mL (41% vs 20%); while the number of patients aged ≥ 65 years (49% vs 90%) favored the cabozantinib arm of the CELESTIAL study compared with our study [13]. Post-hoc subgroup analysis of the CELESTIAL study, however, consistently showed very low 24-week PFS rates in every placebo subgroup with ≥ 10 patients. A second post-hoc analysis was conducted by random sampling of 20 patients’ data from the CELESTIAL study so that the population had the same distribution as the prior sorafenib cohort of this study of the following four baseline factors: extrahepatic spread and/or macrovascular invasion (Y/N), ECOG performance status (0/ > 0), AFP level (< 400/ ≥ 400 ng/mL) and HCC etiology of nonalcoholic steatohepatitis (Y/N). The mean (2.5th and 97.5th percentiles) of 10,000 sampling results for PFS rate at 24 weeks was 14.6% (9.8–19.6%) and 41.7% (36.6–46.7%) in the placebo and cabozantinib arm, respectively, in line with the original analysis. These post-hoc analyses suggest that baseline characteristics are unlikely to have affected the 24-week PFS rate in the CELESTIAL study placebo arm. Therefore, the fact that the lower limit of the 90% CI of the PFS rate at 24 weeks in the prior sorafenib cohort of this study exceeded the pre-specified threshold of 11.1% (which was the 24-week PFS rate in the placebo group of the CELESTIAL study) indicates that the efficacy of cabozantinib in the Japanese patient population of our study is a real effect despite the absence of a concurrent control arm.

Currently approved second-line treatment options for advanced HCC post-sorafenib in Japan are regorafenib and ramucirumab. However, the study designs of the registration trials for these agents had more restrictive patient eligibility criteria than the present study. The RESORCE phase 3 trial of regorafenib excluded patients who were intolerant to sorafenib [14], while the population of the REACH-2 study of ramucirumab was limited to patients with a baseline AFP level ≥ 400 ng/mL [15]. Data from this study, therefore, provide reassurance that cabozantinib is an effective treatment for Japanese patients with sorafenib-treated HCC, including patients with two lines of prior treatment, and irrespective of sorafenib tolerability and baseline AFP levels.

Lenvatinib use is becoming more widespread in Japan since its recent approval as a first-line treatment option for unresectable HCC [7]. However, there are no published prospective clinical studies for second-line agents following lenvatinib failure or intolerance and, consequently, no approved treatments for this population at present. It is notable that more patients in the sorafenib arm of the REFLECT phase 3 trial received post-study medication than in the lenvatinib arm (39% vs. 33%) [7]. In the absence of new data, a lack of approved second-line options is likely to become a larger problem in the near future as lenvatinib utilization increases, and as other first-line treatment options, such as atezolizumab–bevacizumab combination therapy, become available [16]. To reflect the new clinical reality, this study included a cohort of patients previously treated with agents other than sorafenib, including lenvatinib, and inhibitors of programmed cell death protein 1 (PD-1) and programmed cell death ligand 1/2 (PD-L1/L2) for an exploratory evaluation. Although only a small number of patients were included, the observed efficacy was promising and comparable with prior sorafenib-treated patients in the CELESTIAL study (PFS rate at 24 weeks: 16.7% vs. 38.4%; OS rate at 6 months: 79% vs. 72%). PFS rate at 24 weeks was lower in the sorafenib-naïve cohort compared with the prior sorafenib cohort in this trial. These two cohorts differed in terms of HCC etiology, baseline metastatic disease burden, performance status, and AFP levels, factors that are generally considered to influence the effectiveness of HCC treatment. However, in the CELESTIAL trial, there was no significant association between those baseline characteristics and 24-week PFS rate with cabozantinib, as indicated by similar rates for all subgroups as for the entire cabozantinib-treated group (unpublished data). Therefore, further studies are required to determine whether the difference in PFS rate at 24 weeks between the sorafenib-naïve and prior sorafenib cohorts in this trial is a real effect related to prior treatment, or merely an artefact of the relatively small number of patients evaluated.

AEs associated with cabozantinib in this study were generally manageable with dose modifications (including dose reduction and interruption) and supportive care in both the prior sorafenib and sorafenib-naïve cohorts. Dose reduction rates in response to AEs were relatively high in this study at 91.2%. Nevertheless, safety outcomes were consistent with those in the CELESTIAL study, and no new safety concerns were observed in Japanese patients [13]. Rates of AESIs related to antiangiogenic therapy were also comparable to the CELESTIAL study and with previous studies of cabozantinib in patients with renal cell carcinoma [13, 17], with the exception of one patient with a serious AE of hepatic encephalopathy. It is notable that cabozantinib showed a manageable safety profile despite the inclusion of five patients in the prior sorafenib cohort who ended sorafenib treatment because of intolerance. This is pertinent because these five patients would not be eligible for second-line treatment with regorafenib.

As in the CELESTIAL trial, the protocol for this study allowed for flexible dosing to help manage AEs while maximizing study drug exposure once events had resolved. A previous exposure–response analysis has shown a greater OS and PFS benefit in advanced HCC with cabozantinib 60 or 40 mg/day compared with a 20 mg/day dose [18]. Conversely, AEs of PPE syndrome, diarrhea, and hypertension are lower at cabozantinib 20 or 40 mg/day relative to the 60 mg/day dose [18]. Cabozantinib plasma concentrations fell over the course of this study, reflecting dose modifications. However, median cabozantinib plasma concentrations at Week 9 in both cohorts were comparable with those in the much larger-scale CELESTIAL study [19]. The data suggest that cabozantinib exposures in Japanese patients were comparable with those in the CELESTIAL study.

This study was designed to verify the findings of CELESTIAL in a Japanese advanced HCC population. As such, the number of patients enrolled in the prior sorafenib cohort provided sufficient statistical power to assess the primary objective. However, it is acknowledged that a larger patient number and a longer study duration may provide useful additional clinical data such as OS and potential differential effect of cabozantinib in various prior treatment subgroups. The analysis of the sorafenib-naïve cohort was exploratory in nature and, although promising efficacy with cabozantinib was observed, the small number of patients, the relatively short follow-up period, and the absence of a control arm prevent clinical conclusions from being drawn. Larger scale controlled studies of cabozantinib in sorafenib-naïve patients with HCC are warranted.

In summary, the effectiveness and manageable safety profile demonstrated for cabozantinib 60 mg/day in this study indicates that it has a favorable benefit/risk profile as second- or third-line treatment for Japanese patients with advanced HCC who have previously received sorafenib. This includes patients who are resistant or intolerant to sorafenib, and is irrespective of baseline AFP levels.

## Supplementary Information

Below is the link to the electronic supplementary material.

Supplementary file1 (PDF 214 KB)

Supplementary file2 (DOCX 15 KB)

Supplementary file3 (DOCX 13 KB)

## Abbreviations

AE: Adverse events
AFP: Alpha-fetoprotein
CI: Confidence interval
CR: Complete response
DCR: Disease control rate
ECOG: Eastern Cooperative Oncology Group
HCC: Hepatocellular carcinoma
ORR: Objective response rate
OS: Overall survival
PD: Progressive disease
PFS: Progression-free survival
PPE: Palmar–plantar erythrodysesthesia
PR: Partial response
QD: Once daily
SD: Stable disease
VEGF: Vascular endothelial growth factor
