# Supplementary material for: Cabozantinib in Japanese patients with advanced hepatocellular carcinoma: a phase 2 multicenter study
Source: J Gastroenterol. 2021 Jan 3;56(2):181–90. doi: 10.1007/s00535-020-01753-0 (PMC7862203; doi:10.1007/s00535-020-01753-0)
Supplement: Supplementary file 1 — Supplementary file1 (PDF 214 KB) [file 535_2020_1753_MOESM1_ESM.pdf]

Supplementary Figure 1

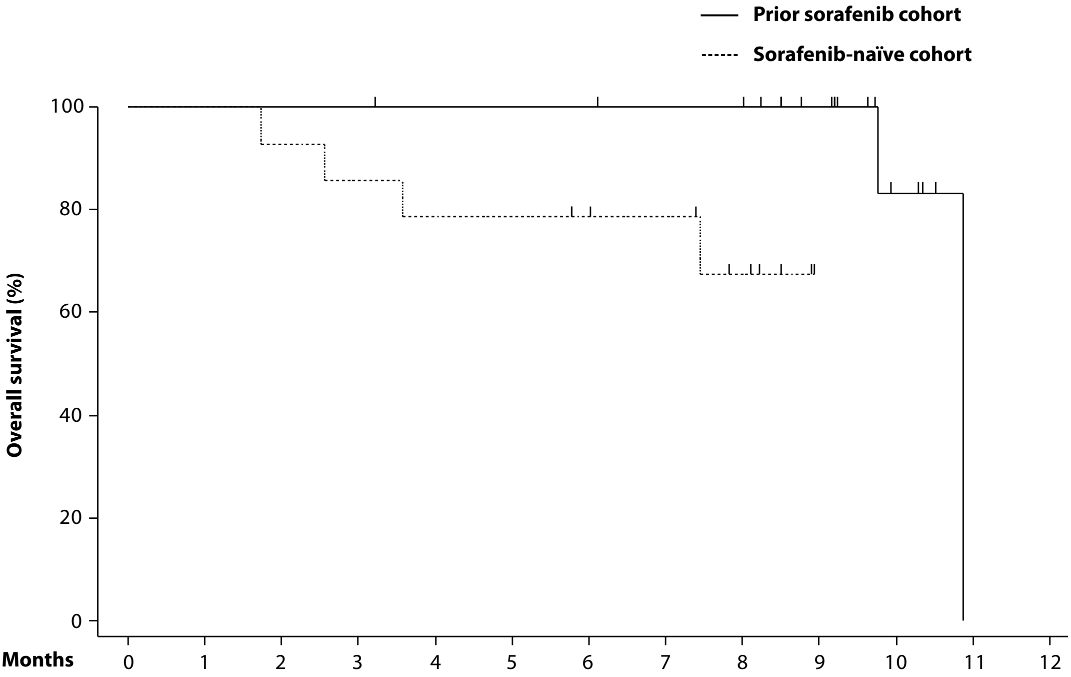

Numbers at risk											
**Prior sorafenib cohort**	n=20	n=20	n=20	n=20	n=19	n=19	n=19	n=18	n=18	n=12	n=4
**Sorafenib-naïve cohort**	n=14	n=14	n=13	n=12	n=11	n=11	n=9	n=8	n=5		
