# Supplementary material for: Cabozantinib in Japanese patients with advanced hepatocellular carcinoma: a phase 2 multicenter study
Source: J Gastroenterol. 2021 Jan 3;56(2):181–90. doi: 10.1007/s00535-020-01753-0 (PMC7862203; doi:10.1007/s00535-020-01753-0)
Supplement: Supplementary file 2 — Supplementary file2 (DOCX 15 KB) [file 535_2020_1753_MOESM2_ESM.docx]

**Supplementary Table 2. Adverse events of special interest (safety analysis set)**

| n (%) | **Prior sorafenib (n=20)** | | **Sorafenib-naïve (n=14)** | | **Total (n=34)** | |
| --- | --- | --- | --- | --- | --- | --- |
|  | **Any grade** | **Grade ≥3** | **Any grade** | **Grade ≥3** | **Any grade** | **Grade ≥3** |
| Gastrointestinal perforation | 1 (5.0) | 1 (5.0) | 0 | 0 | 1 (2.9) | 1 (2.9) |
| Fistula | 0 | 0 | 0 | 0 | 0 | 0 |
| Abscess-all  Intra-abdominal and pelvic abscess | 1 (5.0)  1 (5.0) | 1 (5.0) 1 (5.0) | 0 0 | 0 0 | 1 (2.9) 1 (2.9) | 1 (2.9) 1 (2.9) |
| Hemorrhage (Grade ≥3) | – | 0 | – | 0 | – | 0 |
| Thrombotic events  Arterial thrombotic events  Venous and mixed/unspecified thrombotic events | 0 0 0 | 0 0 0 | 1 (7.1) 0 1 (7.1) | 0 0 0 | 1 (2.9) 0 1 (2.9) | 0 0 0 |
| Wound complication | 0 | 0 | 0 | 0 | 0 | 0 |
| Hypertension | 9 (45.0) | 4 (20.0) | 7 (50.0) | 4 (28.6) | 16 (47.1) | 8 (23.5) |
| Osteonecrosis | 0 | 0 | 0 | 0 | 0 | 0 |
| Palmar-plantar erythrodysethesia syndrome | 16 (80.0) | 4 (20.0) | 10 (71.4) | 5 (35.7) | 26 (76.5) | 9 (26.5) |
| Proteinuria | 3 (15.0) | 2 (10.0) | 4 (28.6) | 1 (7.1) | 7 (20.6) | 3 (8.8) |
| Reversible posterior leukoencephalopathy syndrome | 0 | 0 | 0 | 0 | 0 | 0 |
| Diarrhea | 15 (75.0) | 1 (5.0) | 6 (42.9) | 0 | 21 (61.8) | 1 (2.9) |
| QT prolongation | 0 | 0 | 0 | 0 | 0 | 0 |
| Renal failure | 1 (5.0) | 0 | 1 (7.1) | 1 (7.1) | 2 (5.9) | 1 (2.9) |
| Hypothyroidism | 7 (35.0) | 0 | 3 (21.4) | 0 | 10 (29.4) | 0 |
| Hepatotoxicity | 2 (10.0) | 1 (5.0) | 2 (14.3) | 0 | 4 (11.8) | 1 (2.9) |
