# Supplementary material for: Cabozantinib in Japanese patients with advanced hepatocellular carcinoma: a phase 2 multicenter study
Source: J Gastroenterol. 2021 Jan 3;56(2):181–90. doi: 10.1007/s00535-020-01753-0 (PMC7862203; doi:10.1007/s00535-020-01753-0)
Supplement: Supplementary file 3 — Supplementary file3 (DOCX 13 KB) [file 535_2020_1753_MOESM3_ESM.docx]

**Supplementary Table 1. Key prior HCC therapy (full analysis set)**

|  | **Prior sorafenib (n=20)** | **Sorafenib-naïve (n=14)** | **Total (n=34)** |
| --- | --- | --- | --- |
| Cessation of sorafenib therapy due to intolerance, n (%) | 5 (25.0) | – | 5 (14.7) |
| Median total duration of prior lenvatinib treatment for HCC, months (range) | 2.2 (0.1–4.4) | 3.5 (1.3–6.5) | 3.5 (0.1–6.5) |
| Median time from progression after most recent prior systemic non-radiation anticancer regimen for HCC to enrollment, months (range) | 0.95 (0.5–28.3) | 1.08 (0.3–3.4) | 1.08 (0.3–28.3) |

HCC, PD-1, programmed cell death protein 1; PD-L1/L2, programmed cell death-ligand 1/2.
